# GPS-Free, Error Tolerant Path Planning for Swarms of Micro Aerial Vehicles with Quality Amplification ‡

**DOI:** 10.3390/s21144731

**Published:** 2021-07-10

**Authors:** Michel Barbeau, Joaquin Garcia-Alfaro, Evangelos Kranakis, Fillipe Santos

**Affiliations:** 1 School of Computer Science, Carleton University, Ottawa, ON K1S 5B6, Canada; 2 Telecom SudParis, Institut Polytechnique de Paris, 91120 Palaiseau, France; 3 Institute of Computing, University of Campinas, 13083-852 Campinas, Brazil

**Keywords:** micro aerial vehicles (MAVs), autonomous aerial vehicles, MAV swarm, goal location, quadcopters, information sharing, localization, location, path planning

## Abstract

We present an error tolerant path planning algorithm for Micro Aerial Vehicle (MAV) swarms. We assume navigation without GPS-like techniques. The MAVs find their path using sensors and cameras, identifying and following a series of visual landmarks. The visual landmarks lead the MAVs towards their destination. MAVs are assumed to be unaware of the terrain and locations of the landmarks. They hold a priori information about landmarks, whose interpretation is prone to errors. Errors are of two types, *recognition* or *advice*. Recognition errors follow from misinterpretation of sensed data or a priori information, or confusion of objects, e.g., due to faulty sensors. Advice errors are consequences of outdated or wrong information about landmarks, e.g., due to weather conditions. Our path planning algorithm is cooperative. MAVs communicate and exchange information wirelessly, to minimize the number of recognition and advice errors. Hence, the quality of the navigation decision process is amplified. Our solution successfully achieves an adaptive error tolerant navigation system. Quality amplification is parameterized with respect to the number of MAVs. We validate our approach with theoretical proofs and numeric simulations.

## 1. Introduction

Micro Aerial Vehicles (MAVs) are a popular type of drones. They are equipped with sensors and cameras, enabling hovering and navigation over complex three-dimensional terrains. They are used in a variety of applications, including sewer inspection [1], search and rescue operations [2] and parcel delivery [3]. Large terrains can be covered by so called swarms, namely collaborative teams of MAVs that exchange information gathered during navigation. They are required to be resilient to failures of all kinds, such as during navigation or due to sensor malfunctions. We are interested in designing swarm algorithms that are resilient in the presence of failures.

**Figure 1:**
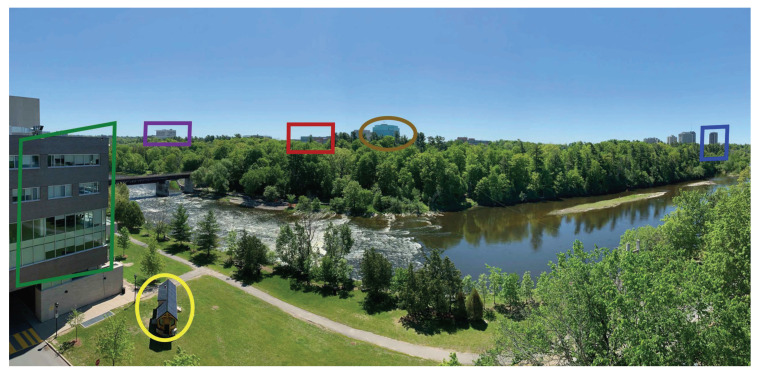
Sample picture taken from an aerial vehicle, together with the identification of six landmarks at the campus of Carleton University.

We present an error tolerant path planning algorithm for MAV swarms. We assume MAV navigation without using any GPS-like technique. MAVs find their way using sensors and cameras, in order to identify and follow a series of visual landmarks. The visual landmarks lead the MAVs towards the destinations. We assume that the MAVs are unaware of the terrain and locations of the landmarks. Figure 1 shows the idea. A number of landmarks are highlighted. A swarm of MAVs collectively identify a series of such landmarks over an inspected terrain. The identification of landmarks determines paths that must be followed.

Swarms fly over terrains comprising multiple landmarks. The landmarks also include the starting and terminal points of a well-defined path. The MAVs may hover over the landmarks either on their own or in formation. They may hop from any one landmark to any other. The landmarks are identified as vertices. The resulting system forms a complete graph. Recall that the MAVs are unaware of the terrain and locations of the landmarks. However, they have the capability to visually recognize them. Furthermore, the MAVs may communicate and exchange information wirelessly as long as they are within communication range of each other. The MAVs are required to find a flight path from the starting point, leading to the terminal point.

We assume that the MAVs hold information about landmarks. Interpretation of this information is error prone. We consider two types of errors: recognition and advice. Recognition errors are due to misinterpretation of sensed data or a priori information, or confusion of objects, e.g., due to faulty sensors. Advice errors follow from changing or wrong information associated with landmarks, e.g., due to weather conditions. Path planning builds upon swarm cooperation. MAVs communicate and exchange information wirelessly, with the aim to reduce the amount of recognition and advice errors. Collaboratively exchanging information, the MAVs amplify the quality of decisions pertaining to the navigation process. The swarm gets equipped with an adaptive error tolerant navigation, where the degree of quality is related to the number of participating MAVs.

We show that the approach augments the probability of navigation correctness proportionally to the number of MAVs in a swarm, when wireless communications allow cooperation and exchange of information about landmarks. Indeed, single MAV navigation is directly affected by recognition and advice errors. The MAV can get disrupted and lost. With an increasing number of MAVs in a swarm, communications and exchange of information take place. Quality of sensor fusion increases. We analyze the reduction of the error probability induced by this algorithm. Quality amplification is demonstrated both analytically and with simulation.

Section 2 reviews related work. Section 3 and Section 4 present our navigation algorithm. Section 5 evaluates the work. Section 6 concludes the paper.

## 2. Related Work

Surveys on path planning algorithms for unmanned aerial vehicles have been authored by Goerzen et al. [4] and Radmanesh et al. [5]. Several algorithms build on solutions originally created for computer networks. Some of the proposed solutions leverage algorithms created in the field of classical robotics, such as approaches using artificial potential functions [6], random trees [7], or Voronoi diagrams [8]. Path planning may be addressed in conjunction with team work and formation control [9]. There are ideas that have been tailored specifically to quadcopters [10]. Other constraints that the MAVs in the swarm must satisfy include the capability of each MAV to match the velocity of its neighbors in the swarm, as well as staying close to the neighbors while addressing collision avoidance. Recent works have addressed these issues applying Lyapunov theory [11,12] and attraction–repulsion functions [13,14].

Our research is closely related to works on navigation using topological maps [15]. Navigation does not rely on coordinates. The MAVs find their way recognizing landmarks. Weinstein et al. [16] propose the use of visual odometry as an alternative localization technique to, e.g., GPS-like techniques. The idea is as follows. The MAVs use their onboard cameras (e.g., downward facing cameras), combined by some inertial sensors, to identify and follow a series of visual landmarks. The visual landmarks lead the MAV towards the target destination. Unlike GPS, the technique allows the MAV to operate without boundaries in both indoor and outdoor environments. No precise information about concrete visual odometry techniques are reported by Weinstein et al. in their work. However, some ideas can be found in [15,17].

Maravall et al. [15,17] propose the use of probabilistic knowledge-based classification and learning automata for the automatic recognition of patterns associated with the visual landmarks that must be identified by the MAVs. A series of classification rules in their conjunctive normal form (CNF) are associated with a series of probability weights that are adapted dynamically using supervised reinforcement learning [18]. The process is conducted using a two-stage learning procedure. During the first process, a series of variables are associated with each rule. For instance, the variables associated with the construction of a landmark recognition classifier are constructed using images’ histogram features, such as standard deviation, skewness, kurtosis, uniformity and entropy. During the second process, a series of weights are associated with every variable. Weights are obtained by applying a reinforcement algorithm, i.e., incremental R-L algorithm in [15,18], over a random environment. As a result, the authors obtain a specific image classifier for the recognition of landmarks, which is then loaded to the MAVs.

The resulting classifiers had been tested via experimental work. MAVs with high-definition cameras, recording images at a resolution of 640 × 360 pixels, at the speed of 30 fps (frames per second) are loaded a given classifier, to evaluate a visual classification ratio. Each experiment consists of building a classifier and getting the averaged ratio. Results by Maravall et al. in [17,19] show an average empirical visual error ratio of about 20% (i.e., 80% chances of properly identifying the landmarks, on average). The results are compared to some other well-established pattern recognition methods for the visual identification of objects, such as minimum distance and *k*-nearest neighbor classification algorithms. The previous contribution is complemented by Fuentes et al. and Maravall, et al. in [15,20], by combining the probabilistic knowledge-based classifiers with bug algorithms [8], to provide the MAVs with a navigation technique to traverse a visual topological map composed of several visual landmarks. A technique is used to compute the entropy of the images captured by the MAV, in case a decision must be made (e.g., to decide whether to go go in a south or north direction). The idea is as follows. The MAV uses the camera onboard and takes images from several directions. Afterward, it processes the images to chose a given direction. The lower the entropy of a captured image, the lower the probability of going towards an area containing visual landmarks. Conversely, the higher the entropy of a captured image, the higher the probability of going towards an area surrounded by landmarks. Using this heuristic, the MAV collects candidate images with maximum entropy (e.g., by driving the MAV forward and backward some meters) prior executing a bug algorithm to locate the landmarks [15].

The main contribution of our work is the introduction of a new MAV swarm path planning algorithm building upon drone-to-drone communication and collaboration. The algorithm does not require the use of GPS localization technology. Path planning and navigation use solely a priori provided visual facts and real-time visual data analysis. We acknowledge that provided facts may be wrong and that visual data analysis may fail. The impacts of wrong facts or analysis failures are mitigated by drone-to-drone communication, exchange of information and a majority-based decisional framework. The algorithm performance versus recognition and advice error rates is analyzed in depth. We demonstrate that, in this setting, when the number of MAVs in a swarm increases, the quality of the decision process is amplified, thanks to the exchange of information collected by the individual MAVs. We also look into the energy cost of this process with respect to the number of MAVs in a swarm and advice recognition rates.

## 3. Error Prone Navigation

**Figure 2:**
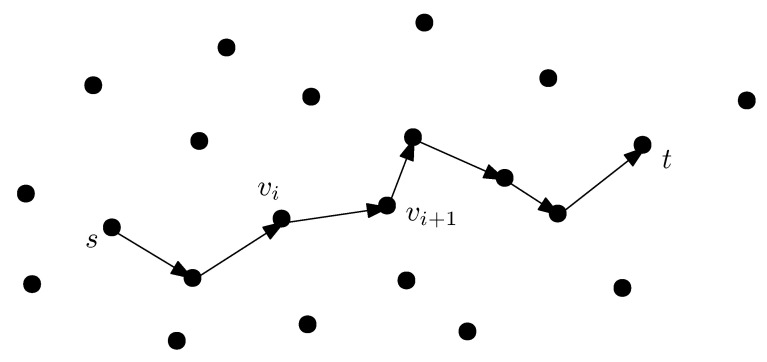
Flight path from source *s* to destination *t*. Edge $\left(v_{i},v_{i+1}\right)$ is an intermediate segment connecting landmarks $v_{i}$ and $v_{i+1}$.

We identify the landmarks with the *n* vertices of a complete graph G=(V,E). Starting at *s* and ending at *t*, the MAVs are seeking flight path connecting k+1 vertices

$$s:=v_{0},v_{1},\ldots ,v_{i},v_{i+1},\ldots ,v_{k}:=t$$

where $v_{0},v_{1},\ldots ,v_{i},v_{i+1},\ldots ,v_{k}$ are in *V*, see Figure 2. The MAVs have to navigate and find a flight path from *s* to *t* using clues. When hovering over an area, a MAV acquires data through its camera and other sensors, which may be visual, acoustic, etc. These data are used for landmark searching. A priori, the MAVs are given clues and specific characteristics about the landmarks. For example, the MAVs may be seeking a green door or a tall building.

The landmarks provided have a priori information whose interpretation (by the MAVs) is prone to errors. We distinguish two types of errors, namely, recognition and advice. Recognition errors are due to misinterpretation of sensed data and a priori information or confusion of objects. For example, a MAV has found a green door which in fact is not a door but rather a window. The recognized object is incorrect. We assume that, for some real number *p* in the interval [0,1], the value *p* is the probability that a MAV performs recognition erroneously and 1−p that it is correct.

Advice errors about landmarks occur because the information provided is not up to date or even wrong. For example, upon finding a landmark, a MAV is advised to traverse a certain distance within the terrain in a northern direction where it will find the next landmark, say a restaurant, but this information is wrong because the restaurant is no longer there. We assume that, for some real number *q* in the interval [0,1], the value *q* is the probability that the advice provided to a MAV about a landmark is invalid or erroneously interpreted and 1−q that it is valid and correctly interpreted.

Recognition and advice errors are independent of each other. An important point to be made is that we assume that recognition and advice are random processes. For all MAVs, we make the assumption that recognition errors are independent and identically distributed and advice errors are also independent and identically distributed. The MAVs act independently of each other. Moreover, the outcome of the recognition process is random with a probability of success that depends on the parameter *p*. A similar observation applies to the advice process. As a consequence, we can use this to our advantage so as to improve the recognition and advice mechanisms for swarms of MAVs.

Assume a MAV is navigating the terrain through a flight path, denoted as *P*, consisting of *k* vertices $v_{0}:=s,v_{1},\ldots ,v_{i},v_{i+1},\ldots ,v_{k}:=t$ from *s* to *t*. An edge $\left\{v_{i},v_{i+1}\right\}$ corresponding to a segment of flight path *P* is said to be correctly traversed if and only if the advice provided about the landmark associated with vertex $v_{i}$ is valid and correctly interpreted and the landmark associated with vertex $v_{i+1}$ is correctly recognized. For i=0,…,k−1, the flight path *P* is correctly traversed if and only if each of its segments defined by an edge $\left\{v_{i},v_{i+1}\right\}$ is correctly traversed.

At the start, a MAV is given a flight plan. The flight plan defines the flight path *P*. For each vertex $v_{i}$, i=0,…,k−1, the flight plan comprises advice for searching the next landmark, such as directional data. For each vertex $v_{i+1}$, the flight plan contains recognition data, such as landmark characteristics. A flight plan is correctly performed solely if every single segment is correctly traversed.

We obtain the following quantitative characterization of segment correctness and flight path in terms of recognition and advice probabilities.

**Lemma** **1.** *A flight plan leading to a path of length k is correctly performed with probability ${\left(1-p\right)}^{k}{\left(1-q\right)}^{k}$.*

**Proof.**  For individual segments i=0,…,k−1, we have

$$\begin{array}{cc} \mathrm{Pr}[\left\{v_{i},v_{i+1}\right\}\;\mathrm{is}\;\mathrm{correct}] & =\mathrm{Pr}[\mathrm{advice}\;\mathrm{at}\;v_{i}\;\mathrm{and}\;\mathrm{recognition}\\ \mathrm{at}\;v_{i+1}\;\mathrm{are}\;\mathrm{correct}] & =(1-p)(1-q). \end{array}$$

For the whole flight plan for path *P*, we have

$$\begin{array}{cc} \mathrm{Pr}[P\;\mathrm{is}\;\mathrm{correct}] & =\mathrm{Pr}[\forall i\left(\left\{v_{i},v_{i+1}\right\}\;\mathrm{is}\;\mathrm{correct}\right)]\\ \; & =\prod_{i=0}^{k-1}\mathrm{Pr}\left[\left\{v_{i},v_{i+1}\right\}\;\mathrm{is}\;\mathrm{correct}\right]\\ \; & ={\left(1-p\right)}^{k}{\left(1-q\right)}^{k}. \end{array}$$

This proves the lemma.    □

Lemma 1 is valid for a single MAV that is recognizing landmarks and navigating from a start point to a terminal point. In Section 4, it is shown how to improve the probability of correctness for a swarm of co-operating MAVs that communicate and exchange information with each other.

In a swarm, we may take advantage of communications and collaboration among the MAVs so as to amplify the quality of a priori and sensed data. To this end, we use the principle of maximum likelihood.

Algorithms 1 and 2 define the main processes. Algorithm 1 applies majority recognition. Algorithm 2 applies the advice. It should be emphasized that the amplification of recognition and advice, implied by the majority rule used in the two algorithms above, is based on a binary decision. To illustrate this fact, consider the case of amplification of the quality of recognition. First of all, it is assumed that all the MAVs in the swarm run the same visual recognition software. Hence, the set of possible outcomes of the MAVs’ visual systems is partitioned into two mutually disjoint sets. The first set can be interpreted as the container of positive outcomes, the second set as the container of negative outcomes. This is to be the same for all the MAVs. For a binary decision example, consider a swarm of five MAVs which is to decide whether the object viewed is either a Door (D) or a Window (W). If the answers of the individual MAVs are D, W, D, W, D, then the majority output will be Door.

A similar interpretation is being used for the advice algorithm software which is executed by “smart landmarks” giving advice to the MAVs, i.e., providing the direction the swarm should follow next. For a binary example with a swarm of five MAVs, assume that the landmarks may give either the answer North (N) or South (S). If the advice collected by the MAVs are N, S, S, N, N, then the majority decision will be North.
**Algorithm 1** Majority Recognition Algorithm for a swarm of *m* MAVs1:Each MAV performs landmark recognition2:MAVs exchange information3:**if** there is a landmark common to the majority (of at least ⌈m/2⌉ MAVs) **then**4:    the MAV swarm adopts this common landmark5:**else**6:    every MAV adopts its own recognized landmark7:**end if**

**Algorithm 2** Majority Advice Algorithm for a swarm of *m* MAVs
1:Each MAV takes the advice provided for the visited landmark2:MAVs exchange information3:**if** there is a majority advice interpretation (for at least ⌈m/2⌉ MAVs) **then**4:    all MAVs follow this common advice interpretation5:
**else**
6:    the MAVs follow their own advice interpretation7:
**end if**



## 4. Quality Amplification and Error Reduction

### 4.1. Reducing the Error Probability

The collaborative landmark recognition process defined by Algorithm 1 applies to a swarm composed of *m* MAVs. Let $p_{m}$ denote the error probability of the majority rule applied in Algorithm 1; this is given by the following formula:

(1) pm=1−∑i=⌈m/2⌉mmi(1−p)ipm−i

Now, we show that the majority rule improves the error probability *p*.

**Lemma** **2.** *For p<1/2, we have the following inequality:*

(2) 1−p<pm∑i=⌈m/2⌉mmi1p−1i.

**Proof.**  (Lemma 2) The inequality is proved by considering two cases depending on the parity of *m*, the number of MAVs.

**Case 1** (*m* is odd): If *m* is odd, we can express the value as m=2d+1, for some integer d≥1 so that ⌈m/2⌉=d+1. Let $a=\frac{1}{p}-1$ and observe that a>1, since $p<\frac{1}{2}$. From the binomial theorem, we have that

(3) (a+1)m=∑i=0mmiai=∑i=0dmiai+∑i=d+1mmiai=L+U,

where *L* and *U* are defined as follows:

(4) L:=∑i=0dmiai=∑i=0dmd−iad−i, and

(5) U:=∑i=d+1mmiai=∑i=0dmd+i+1ad+i+1.

Now, observe that *L* and *U* have the same number of summands with identical respective binomial coefficients, namely

md−iad−i and md+i+1ad+i+1,

for i=0,1,…,d. In Formulas (4) and (5), observe that the left term when multiplied by $a^{2i+1}$ is equal to the right term, namely a2i+1md−iad−i=md+i+1ad+i+1, for i=0,1,…,d. Since a>1 and d≥1, we conclude that

(6) aL=∑i=0damd−iad−i<∑i=0da2i+1md−iad−i=U.

From Equations (3) and (6), it follows that ${\left(a+1\right)}^{m}=L+U<\left(\frac{1}{a}+1\right)U$.

Since $a+1=\frac{1}{p}$, we conclude that

$$U>\frac{{\left(a+1\right)}^{m}}{\frac{1}{a}+1}=\frac{1-p}{p^{m}}.$$

**Case 2** (*m* is even): The proof is similar to the case when *m* is odd. Since *m* is even, it can be written as m=2d, for some integer d≥1 so that ⌈m/2⌉=d. Let $a=\frac{1}{p}-1$ and observe that a>1, since $p<\frac{1}{2}$. From the binomial theorem, we have that

(7) (a+1)m =∑i=0mmiai=∑i=0d−1miai+∑i=dmmiai=L′+U′,

where $L^{\prime }$ and $U^{\prime }$ are defined as follows:

(8) L′:=∑i=0d−1miai=∑i=1dmd−iad−i, and

(9) U′:=∑i=dmmiai=∑i=0dmd+iad+i.

Now, we compare summands in $L^{\prime }$ and $U^{\prime }$, namely

md−iad−i and md+iad+i,

for i=0,1,…,d. In Formulas (8) and (9), observe that the left term when multiplied by $a^{2i}$ is equal to the right term, namely a2imd−iad−i=md+iad+i, for i=0,1,…,d. Since a>1 and d≥1, we conclude that

(10) aL′≤∑i=0damd−iad−i<∑i=0da2imd−iad−i=U′.

From Equations (7) and (10), it follows that ${\left(a+1\right)}^{m}=L+U<\left(\frac{1}{a}+1\right)U$. Since $a+1=\frac{1}{p}$, we conclude that

$$U^{\prime }>\frac{{\left(a+1\right)}^{m}}{\frac{1}{a}+1}=\frac{1-p}{p^{m}}.$$

Therefore, Inequality (2) is proved in both cases of *m* odd and *m* even. Thus, the proof of Lemma 2 is complete.    □

We may now conclude the following.

**Theorem** **1.** *The majority rule applied to a swarm of m MAVs executing Algorithm 1 reduces the probability of error of the recognition process as long as p is less than 1/2.*

**Proof.**  Let *m* be the number of MAVs. Therefore, $1-p_{m}$ is the probability that the majority is at least composed of ⌈m/2⌉ MAVs correctly performing recognition, i.e.,

(11) 1−pm=∑i=⌈m/2⌉mmi(1−p)ipm−i=pm∑i=⌈m/2⌉mmi1p−1i.

Now, for p<1/2, Lemma 2 says that

(12) 1−p<pm∑i=⌈m/2⌉mmi1p−1i,

which, in view of Equation (11), implies that $p_{m}<p$, i.e., the probability of error for a swarm of *m* MAVs is less than for MAV in solo. This proves the theorem.    □

A similar proof also yields the following.

**Theorem** **2.** *The majority rule applied to a swarm of m MAVs executing Algorithm 2 reduces the probability of error of the advice process as long as q<1/2,*

**Proof.**  The proof is similar to the proof of Theorem 2.    □

Note that there are additional possibilities in Algorithm 2. The MAVs in a swarm could also acquire information either from the same landmark or from different landmarks (although we do not investigate the latter case further).

### 4.2. Approximating the Majority

Let $S_{m}$ be the sum of *m* mutually independent random variables each taking the value 1 with probability *p* and the value 0 with probability 1−p (i.e., Bernoulli random trials). The majority probability discussed above is given by the formula $\mathrm{Pr}[S_{m}\geq \left\lceil \frac{m}{2}\right\rceil ]$. Good approximations of the majority probability for large values of *m* can be obtained from the central limit theorem, which states that

(13) $$\mathrm{Pr}\left[a\leq \frac{S_{m}-mp}{\sqrt{mp(1-p)}}\leq b\right]\to \frac{1}{\sqrt{2\pi }}\int_{a}^{b}e^{-x^{2}/2}dx.\;\mathrm{as}\;m\to \infty$$

(see, for instance [21]). For example, for any *m*, we have that

$$S_{m}\geq \left\lceil \frac{m}{2}\right\rceil \Leftrightarrow \frac{S_{m}-mp}{\sqrt{mp(1-p)}}\geq \frac{\left\lceil \frac{m}{2}\right\rceil -mp}{\sqrt{mp(1-p)}}.$$

Hence, the central limit theorem (13) is applicable with $a=\frac{\left\lceil \frac{m}{2}\right\rceil -mp}{\sqrt{mp(1-p)}}$ and b=+∞, where p<1/2 is a constant.

## 5. Experiments and Simulations

There is an interesting trade-off between the majority probability $p_{m}$ and cost of using a swarm of *m* MAVs. This helps put the probabilistic gains in context w.r.t. the energy consumption and time cost of the swarm.

### 5.1. Cost Measures and Tradeoffs

From Theorem 1, we know that, for any number *m* of MAVs, the error probability *p* is reduced to $p_{m}$, similarly for Theorem 2. We now examine quantitative estimates of this error reduction in relation to specific numbers *m* of MAVs involved.

From Equation (11), observe that we can derive the following identity expressing the ratio of improvement of the probability of correctness:

(14) 1−pm1−p=∑i=⌈m/2⌉mmi(1−p)i−1pm−i

In a way, one can think of the right-hand side of Equation (14) as the “fractional gain” in the correctness probability (because we are employing a majority rule) that improves from 1−p to $1-p_{m}$. In general, we would like on the one hand to ensure that $\frac{1-p_{m}}{1-p}$ is greater than one, i.e., there is effectively a gain, and on the other hand to optimize the right-hand side of Equation (14). Since we are also interested in applying the majority algorithms for a relatively small number of MAVs, we give close form expressions and precise estimates of the optimal value of *p* and the maximum fractional gain, for *m* equal to 2, 3, 4, 5, 6 and 7 MAVs.

**Theorem** **3.** *For m equal to 2, 3, 4, 5, 6 and 7 MAVs, the maximum attainable fractional gain $\frac{1-p_{m}}{1-p}$ and the corresponding error probability p are as listed in the second and third column, respectively, of Table 1.*

**Proof.**  Each of the six cases is examined individually. Recall that the fractional gain is the ratio $\frac{1-p_{m}}{1-p}$ whose expression as given by Equation (14) yields a polynomial which is easily optimized with analytical methods, namely by setting its first derivative equal to zero, or numerical methods.

**Case m=2.** The fractional gain ratio is $\frac{1-p_{2}}{1-p}$, which is equal to $\frac{1-p^{2}}{1-p}$ or the linear polynomial 1+p. It is maximized for *p* equal to 1/2, i.e., the maximum value that can be assumed by *p*. The maximum fractional gain is therefore 1+1/2.

**Case m=3.** The fractional gain ratio is $\frac{1-p_{3}}{1-p}$, which is equal to the polynomial $1+p-2p^{2}$. Setting its derivative equal to 0, we obtain 1−4p=0 and therefore p=1/4. Therefore, the fractional gain attains the maximum value 1+1/8.

**Case m=4.** The fractional gain ratio satisfies $\frac{1-p_{4}}{1-p}=\left(1-p\right)\left(3p^{2}+2p+1\right)$. Differentiating the last polynomial and setting it equal to 0, we obtain $-9p^{2}+2p+-1=0$. Solving the quadratic, we obtain that the fractional gain is maximized when $p=\frac{1+\sqrt{10}}{9}$ and attains the maximum value 1.379.

**Case m=5.** The fractional gain ratio is $\frac{1-p_{5}}{1-p}$, which is equal to the polynomial $10{\left(1-p\right)}^{2}p^{2}+5{\left(1-p\right)}^{3}p+{\left(1-p\right)}^{4}$. Its first derivative is equal to the polynomial $24p^{3}-27p^{2}+2p+1$. One of the roots of this polynomial is 1. We have that $24p^{3}-27p^{2}+2p+1$ is equal to $\left(p-1\right)\left(24p^{2}-3p+1\right)$. The positive root of the quadratic $24p^{2}-3p+1$ is equal to $\frac{3+\sqrt{9+96}}{48}$, or approximately 0.276. The fractional gain ratio attains the maximum value 1.1917.

**Case m=6.** The fractional gain ratio is $\frac{1-p_{6}}{1-p}$, which is equal to the polynomial $1+p+p^{2}+p^{3}-14p^{4}+10p^{5}$. Using the Wolfram Mathematica™ function, solve(diff($1+p+p^{2}+p^{3}-14p^{4}+10p^{5}$),*p*)) we obtain the optimal value

$$p=\frac{6+{\left(38016-1350\sqrt{730}\right)}^{1/3}+3{\left(2\left(704+25\sqrt{730}\right)\right)}^{1/3}}{150}\approx 0.39751\ldots$$

**Case m=7.** The fractional gain ratio is $\frac{1-p_{7}}{1-p}$, which is equal to the polynomial

$${\left(1-p\right)}^{3}\left(1+4p+10p^{2}+20p^{3}\right)$$

Using the Wolfram Mathematica™ function solve(diff((${1-p)}^{3}(1+4p+10p^{2}+20p^{3}$), *p*)), we obtain the optimal value

$$p=\frac{10+{\left(217000-15120\sqrt{190}\right)}^{1/3}+2\left(35\left(775+54\sqrt{(}190\right)\right){)}^{1/3}}{360}\approx 0.29357$$

□

For m=2,…7, Table 2 displays the polynomials modeling the fractional gains and the majority error probability $p_{m}$.

Table 3 displays the optimal error probability, fractional gain and majority error probability $p_{m}$ versus a given number *m* of MAVs, where m≤20.

**Figure 3:**
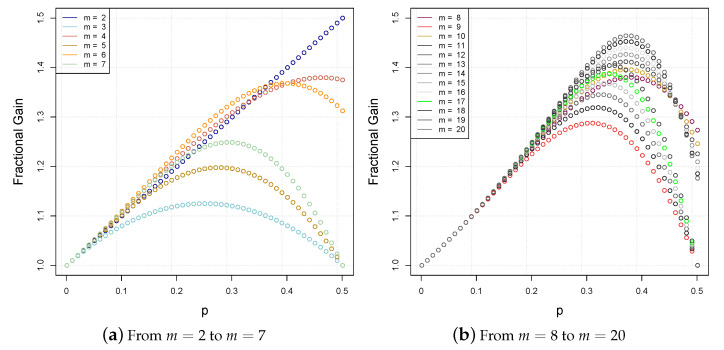
Plots of the fractional gain function $\frac{1-p_{m}}{1-p}$ for varied *p*’s and *m*’s.

Figure 3a plots the evaluation of equation $\frac{1-p_{m}}{1-p}$ from m=2 to m=7. Figure 3b plots the same evaluation, from m=8 to m=20. The curves indicate the maximum fractional gains as a function of the probability *p*.

### 5.2. Numerical Simulations

Algorithms 1 and 2 have been integrated into a Java simulator, which implements swarm populations modeled as mobile agents. Each swarm executes the algorithms within a terrain of interconnected landmarks. It consists of a simple discrete event, time-step based simulation engine, in which the swarm executes our algorithms at every step of simulated time. The simulation engine implements a discrete event scheduler, a graphical view, a data collection system and the simulated objects themselves, i.e., landmarks and agents. Videocaptures and source code are available online [22].

**Figure 4:**
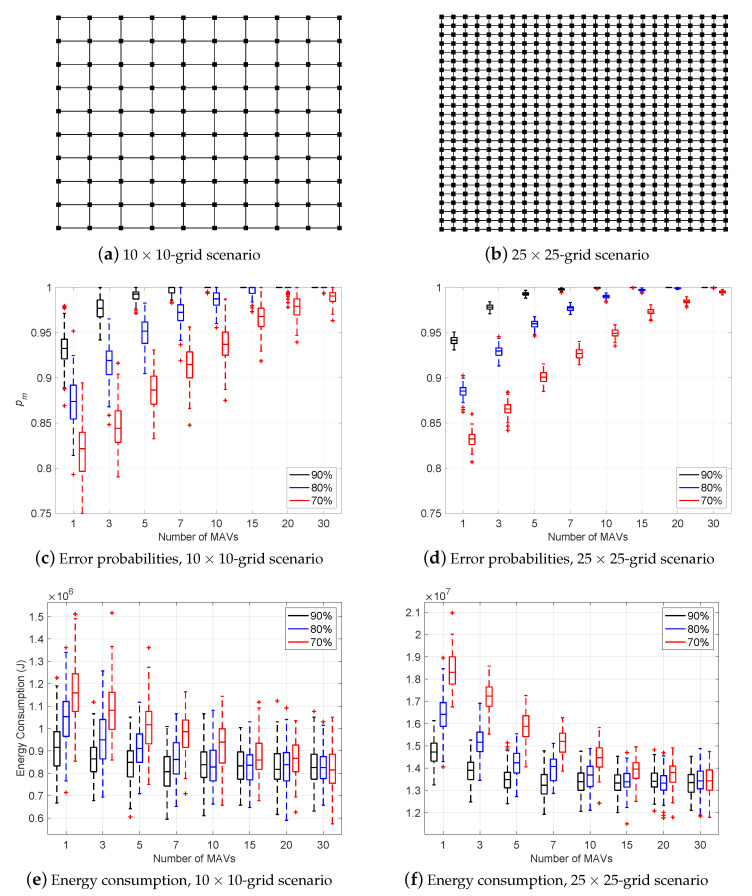
Experimental results for the two first scenarios. (**a**) depicts the 10×10-grid scenario. (**b**) depicts the 25×25-grid scenario. (**c**,**d**) show the relation between the number of MAVs and error probability of the majority rule ($p_{m}$) varying the recognition and advice error ratios between 70% (red), 80% (blue) and 90% (black). The vertical axis represents $p_{m}$. The horizontal axis represents the number of MAVs. (**e**,**f**) show the relation between the number of MAVs vs. energy consumption, when varying the recognition and advice error ratios. The vertical axis represents the energy consumption (joules).

**Figure 5:**
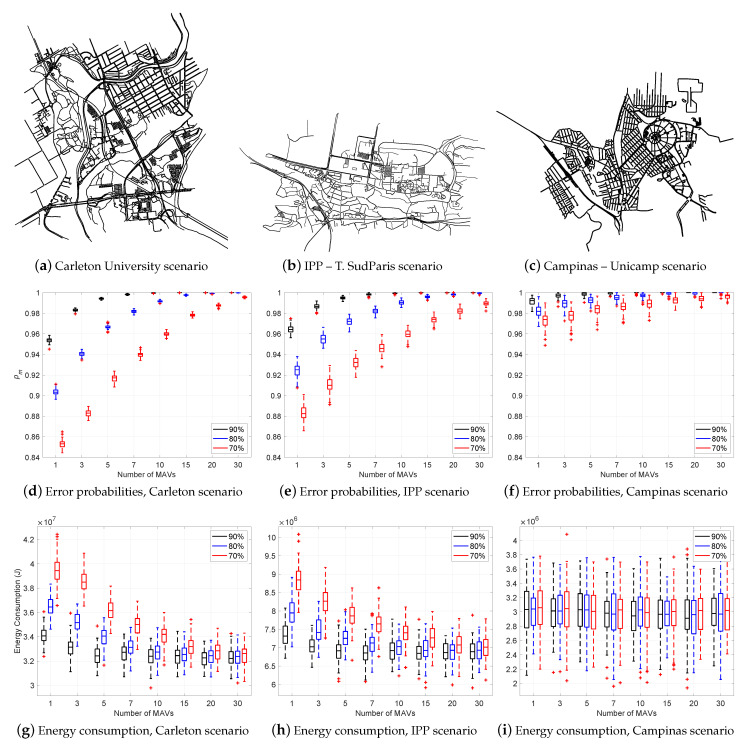
Experimental results for the three last scenarios. (**a**) depicts the Carleton University map scenario. (**b**) depicts the IPP—T. SudParis scenario. (**c**) depicts the Campinas—Unicamp scenario. (**d**–**f**) show the relation between the number of MAVs and error probability of the majority rule ($p_{m}$) varying the recognition and advice error ratios between 70% (red), 80% (blue) and 90% (black). The vertical axis represents $p_{m}$. The horizontal axis represents the number of MAVs. (**g**–**i**) show the relation between the number of MAVs vs. energy consumption, when varying the recognition and advice error ratios. The vertical axis represents the energy consumption (joules).

Using our Java simulation, we validate five different scenarios. Every scenario relates the number of MAVs with the error probability of the majority edasrule varying the recognition and advice error ratios among the percentages 70%, 80% and 90% (cf. Section 3). Figure 4a,b represent two grid structures of MAVSIM, i.e., a 10×10-grid and a 25×25-grid. Figure 5a is a topological structure imported from the OpenStreetMap (OSM (https://www.openstreetmap.org (accessed on 9 July 2021))) project, using *Carleton University* as the location; Figure 5b is a topological structure imported from OSM using Telecom SudParis (at the campus of the Institut Polytechnique de Paris (IPP)) as the location; Figure 5c is a topological structure imported from OSM using the University of Campinas as a location.

### 5.3. Performance Evaluation

Table 4 shows key characteristics for each of the five scenarios. For every scenario, the second column lists the types of landmarks that are present. The third column lists the number of nodes contained in the map. The fourth column specifies the quantity of nodes used as landmarks.

For each of the five scenarios, Figure 4 and Figure 5 (cf. Parts Figure 4c,d and Figure 5d–f) show the relation between the number of MAVs and error probability $p_{m}$ of the majority rule varying the recognition and advice error ratios among the percentages 70%, 80% and 90%. The horizontal axis lists the numbers of MAVs. The vertical axis represents the majority error probability $p_{m}$. For every scenario, there are three curves corresponding to the recognition and advice error percentages 70% (red), 80% (blue) and 90% (black). For each of the five scenarios, Figure 4 and Figure 5 confirm that the majority rule applied to a swarm of *m* MAVs reduces the probability of error of the recognition process. Our solution benefits from swarm cooperation.

### 5.4. Energy Evaluation

To move a distance *d* at speed $s_{i}$, the energy consumed by a straight line flight $E_{fly}$ can be quantified as the integral of the power $P(s_{i})$ as a function of the speed along the time [23]:

(15) $$E_{fly}\left(d,s_{i}\right)=\int_{t=0}^{t=d/s_{i}}P\left(s_{i}\right)dt=P\left(s_{i}\right)\frac{d}{s_{i}}$$

For the five scenarios, Figure 4 and Figure 5 (cf. Parts Figure 4e,f and Figure 5g–i) show the relation between the number of MAVs and energy consumption varying the recognition and advice error ratios among the percentages 70%, 80% and 90%. In each case, the horizontal axis represents the number of MAVs. The vertical axis represents the energy consumption metric. There are three bars corresponding to the recognition and advice error ratios 70% (red), 80% (blue) and 90% (black). The speed is set to 5 m/s. In the first four scenarios, the results show that the energy consumption of the MAVs reduces to approximately 35%, when the number of MAVs increases. This is because the error probability of the majority rules $p_{m}$ decreases as well. On the other hand, the University of Campinas scenario is a sparse graph. The number of landmarks is relatively small. Energy consumption is less sensitive to the number of MAVs.

## 6. Conclusions

We have presented an error tolerant path planning algorithm for MAV swarms. We have assumed a navigation system in which the MAVs find their path by using their on board cameras, by identifying and following a series of visual landmarks. We have assumed landmarks’ a priori information, but for which interpretation by the MAVs is error prone. We have defined two types of errors: (1) recognition errors, e.g., due to faulty sensors that misinterpret the sensed data, and (2) advice errors caused by the landmarks, e.g., due to weather conditions or outdated information. Our solution benefits from swarm cooperation. When the MAVs in a swarm can communicate and exchange information, the recognition and advice error ratios *get minimized to one fourth* at the expense of *increasing the total number of MAVs by twenty*. We validated the proposal via simulations, implemented in Java, available in a companion repository on github [22].

The recognition and advice algorithms presented are based on binary decision-making. An interesting setting worth exploring is non-binary. For example, consider a swarm of five MAVs required to decide its next move based on the majority color of a door, say, among Yellow (Y), Green (G) and Blue (B); in this case, it is assumed that Y is to be a positive outcome while G, B are negative outcomes. On the one hand, if the respective outputs of the visual systems are Y, G, Y, Y, B, then the Yellow Door is considered to be the positive outcome and occurs three out of five times. On the other hand, if the respective outputs of the visual systems are Y, G, Y, B, B, then there is no majority of identical colors. In particular, the majority can be formed by three identical answers. Such situations can be handled using voting schemes and fuzzy logic, which would be the focus of future research.

The basic idea of our algorithms is to enhance the quality of recognition and advice by having multiple MAVs make a decision after exchanging information they have obtained. Naturally, this increases the cost of movement since multiple MAVs will be traveling to a destination. Therefore, it would be interesting to look at trade-offs of the cost of the search that take into account either time or total energy versus the number of MAVs in the swarm for a given budget.

## Figures and Tables

**Table 1:** Maximum fractional gain vs. number of MAVs (*m*).

*m*	Maximum Fractional Gain	Corresponding *p*
2	1 +1 /2	1/2
3	1 + 1/8	1/4
4	1.379	$\frac{1+\sqrt{10}}{9}$
5	1.198	0.276
6	1.368	0.398
7	1.249	0.294

**Table 2:** Fractional gain polynomials and majority error probability $p_{m}$, for m=2 to m=7.

*m*	Fractional Gain Polynomial	$p_{m}$
2	1+p	0.25
3	$-2p^{2}+p+1$	0.15
4	$-3p^{3}+p^{2}+p+1$	0.25
5	$6p^{4}-9p^{3}+p^{2}+p+1$	0.13
6	$10p^{5}-14p^{4}+p^{3}+p^{2}+p+1$	0.179
7	$-20p^{6}+50p^{5}-34p^{4}+p^{3}+p^{2}+p+1$	0.113

**Table 3:** Left to right, the columns provide (1) the number of MAVs, (2) the optimal value (*p*), (3) the fractional gain $\frac{1-p_{m}}{1-p}$ from m=9 to m=20 and (4) the corresponding majority error.

*m*	Optimal Value (*p*)	Fractional Gain	$p_{m}$
8	0.381	1.380	0.144
9	0.307	1.287	0.111
10	0.372	1.396	0.120
11	0.317	1.319	0.103
12	0.370	1.411	0.110
13	0.326	1.345	0.098
14	0.370	1.426	0.101
15	0.333	1.368	0.083
16	0.371	1.440	0.092
17	0.339	1.387	0.084
18	0.373	1.452	0.084
19	0.344	1.404	0.073
20	0.374	1.464	0.077

**Table 4:** Characteristics of each scenario.

Scenario	Landmark Types	# of Nodes	# of Landmarks
10×10-grid	Random nodes	100	1/4 of the nodes
25×25-grid	Random nodes	625	1/4 of the nodes
Carleton University	Traffic signals, junctions, etc.	3511	335
IPP—Telecom SudParis	Crossing paths, tourism viewpoint, etc.	1413	97
Campinas—Unicamp	Crossing paths, round-about, etc.	1559	29

## Data Availability

Not applicable.
